# Shared and organism-specific host responses to childhood diarrheal diseases revealed by whole blood transcript profiling

**DOI:** 10.1371/journal.pone.0192082

**Published:** 2018-01-29

**Authors:** Hannah A. DeBerg, Mussaret B. Zaidi, Matthew C. Altman, Prasong Khaenam, Vivian H. Gersuk, Freddy D. Campos, Iza Perez-Martinez, Mario Meza-Segura, Damien Chaussabel, Jacques Banchereau, Teresa Estrada-Garcia, Peter S. Linsley

**Affiliations:** 1 Benaroya Research Institute at Virginia Mason, Seattle, Washington, United States of America; 2 Infectious Diseases Research Unit, and Pediatric Emergency Department, Hospital General O’Horan, Mérida, Yucatán, Mexico; 3 Department of Epidemiology and Biostatistics, Michigan State University, Lansing, Michigan, United States of America; 4 Department of Medicine, University of Washington School of Medicine, Seattle, Washington, United States of America; 5 Department of Molecular Biomedicine, CINVESTAV-IPN, México DF, México; 6 The Jackson Laboratory for Genomic Medicine, Farmington, Connecticut, United States of America; Tulane University, UNITED STATES

## Abstract

Globally, diarrheal diseases are a leading cause of death in children under five and disproportionately affect children in developing countries. Children who contract diarrheal diseases are rarely screened to identify the etiologic agent due to time and cost considerations associated with pathogen-specific screening and hence pathogen-directed therapy is uncommon. The development of biomarkers to rapidly identify underlying pathogens could improve treatment options and clinical outcomes in childhood diarrheal diseases. Here, we perform RNA sequencing on blood samples collected from children evaluated in an emergency room setting with diarrheal disease where the pathogen(s) present are known. We determine host response gene signatures specific to *Salmonella*, *Shigella* and rotavirus, but not *E*. *coli*, infections that distinguish them from each other and from healthy controls. Specifically, we observed differential expression of genes related to chemokine receptors or inflammasome signaling in *Shigella* cases, such as CCR3, CXCR8, and NLRC4, and interferon response genes, such as IFI44 and OASL, in rotavirus cases. Our findings add insight into the host peripheral immune response to these pathogens, and suggest strategies and limitations for the use host response transcript signatures for diagnosing the etiologic agent of childhood diarrheal diseases.

## Introduction

Diarrheal diseases are a major global health challenge that disproportionately affect children in developing countries [1–3]. In Mexico, diarrheal diseases are a leading cause of death in children under five [4, 5]. Among this age group, the state of Yucatan has one of the highest incidence rates of diarrhea nationwide (26,618 cases/100,000) [4, 6]. Despite their morbidity and mortality, diarrheal diseases in children are routinely diagnosed and managed based on clinical observations rather than pathogen-specific tests. However, clinical diagnosis and risk stratification is inaccurate and there is a need for straightforward, rapidly identifiable and inexpensive biomarkers that more accurately predict underlying pathogenesis. Historically, a combination of stool culture, serologic tests, and RT-PCR tests have been required to diagnose the cause of illness [7]. Recently, molecular diagnostic tests for detecting pathogens in stool samples have been developed and blood-based gene expression signatures designed to discriminate between bacterial and viral infections have been developed, but high costs have limited their use in resource-poor settings. [7–11]. In a broader context, blood transcript profiling has provided mechanistic insight into a variety of diseases, including infectious disease, cancer, cardiovascular disease, and autoimmune disease [12–14]. The exquisite sensitivity and specificity of whole blood transcript profiling in other studies suggests that it may also offer the potential to determine infectious etiology of diarrheal diseases by serving as a sensor for host peripheral immune responses.

We hypothesized that pathogens causing diarrheal diseases would elicit distinct patterns of gene expression in host peripheral blood that could be used to characterize immune responses associated with each pathogen and with disease severity. To test this hypothesis, we performed RNA sequencing (RNA-seq) on whole blood samples collected from children with acute diarrheal illness, within 8 days of disease onset, as well as from healthy control (HC) subjects. Transcript profiling by RNA-seq allows for quantitative, unbiased analysis of all expressed genes. Here we present results on whole blood transcript signatures from children infected with pathogens causing diarrheal disease, including *Escherichia coli*, *Salmonella* species, *Shigella* species, and rotavirus serotypes. Relative to HC, we observed both pathogen-non-specific and pathogen-specific changes in host gene expression that provide mechanistic insight into the pathogenesis of these diseases, and suggest potential organism-specific biomarkers.

## Materials and methods

### Study design

Clinical studies were conducted from 2010 to 2014 at the Hospital General O’Horan, a major referral hospital in the state of Yucatan, Mexico. Children under 10 years of age seeking medical care at the Pediatric Emergency Room for acute community-acquired diarrhea (less than 8 days) were invited to participate. Patients with underlying gastrointestinal or immunological disorders, including recent GI surgery, were excluded from the study. Healthy, age-matched controls were children visiting the clinic for routine vaccinations or simple fractures. On admission, a trained nurse applied a standardized questionnaire to collect demographic and clinical data. Stool and blood samples were collected from each child on or shortly after admission. Stools were collected in two sterile containers; one contained Cary-Blair media for isolation of bacterial pathogens and the other with no transport media was used for detection of rotavirus. Whole blood was collected in EDTA and Tempus tubes. EDTA tubes were sent to the routine clinical laboratory for complete blood counts. Tempus tubes were frozen at -20 °C and shipped to the Benaroya Research Institute on dry ice. Stool and blood samples were transported to the laboratory within 1 hour. Each individual was assigned a severity category based on a modification of the World Health Organization criteria as described previously [15].

### Laboratory procedures

All stool samples were inoculated onto XLD (*Salmonella* and *Shigella* detection), Hektoen Enteric (*Salmonella* and *Shigella* detection), Brilliant Green (*Salmonella* detection), Cefex (*Campylobacter* detection), TCBS (*Vibrio* detection) and MacConkey (*E*. *coli* detection) agar, as well as tetrathionate (*Salmonella* detection), Rappaport (*Salmonella* detection) and alkaline peptone (*Vibrio* detection) broths and incubated at 37°C for 18–24 h. Identification of *Salmonella* and *Shigella* isolates were performed with conventional biochemical tests and confirmed with API 20E strips (BioMerieux, Marcy l’Etoile, France). It is possible that we excluded some subjects with diarrhea based on a negative culture test that could have been detected with a qPCR assay. Excluding these subjects would decrease the power of our study, however, we do not expect the exclusion of these cases to affect our results as culture tests combined with clinical symptoms are a strong indicator of disease. Five *E*. *coli*-like colonies were selected from each MacConkey agar plate, confirmed with conventional biochemical tests and subjected to PCR testing for diarrheagenic *E*. *coli* (DEC) loci that identified all six reported diarrheagenic *E*. *coli* pathogroups [16]. Rapid detection of rotavirus was performed with a latex agglutination test (Pastorex Rotavirus, BioRad, Hercules, USA). One g aliquots of stool samples were stored at -70 °C and later processed for rotavirus detection by ELISA (Premier Rotaclone, Meridian Bioscience Inc., Cincinnati, USA) and PCR.

### PCR assays for the identification of diarrheagenic *E*. *coli*

All *E*. *coli* strains were further characterized for the presence of DEC group defining loci by previously described PCR assays [17–19]. For all PCR assays, bacterial lysates were prepared by resuspending a single colony in 1 ml of deionized water (MilliQ) in a polypropylene tube, followed by boiling for 1 min and then 2μl of bacterial lysate was used in each PCR assay.

### PCR assays for rotavirus

Stools were diluted 1:10 in sterile water. Three hundred μl of the supernatant was collected, boiled for 10 min and centrifuged. RNA was extracted using the QIAGEN QIAmp MiniElute Virus Spin kit. Reverse transcription was performed with the Invitrogen Superscript III First-Strand Synthesis System to generate cDNA. Next, 3 μl of cDNA was added to 17 μl of PCR Mastermix using the Seeplex Diarrhea ACE Detection Kit (Seegene) according to the manufacturer’s instructions. PCR products were visualized with 1.5% agarose gels stained with ethidium bromide.

### Whole blood RNA sequencing

Whole blood was collected in Tempus reagent at the clinical site at a 1:2 blood:Tempus ratio (Ambion, MA). Total RNA was isolated from whole blood samples using MagMax for Stabilized Blood Tubes RNA Isolation Kit (Ambion, MA) and RNA samples were globin-reduced with GLOBINclear (Ambion, MA) according to manufacturer’s instructions. Libraries were constructed from globin-reduced RNA using the Illumina TruSeq RNA Sample Preparation kit v2 according to manufacturer’s instructions. Libraries were clustered on a flowcell using the TruSeq Single Read Cluster Kit v4, followed by single-read sequencing for 50–58 cycles on a HiSeq2500 sequencer (Illumina, CA).

### RNA sequencing alignment and quality control

Base-calling was performed automatically by Illumina real time analysis software and demultiplexing was performed on Illumina BaseSpace after sequencing to generate FASTQ files; FASTQ reads were trimmed in a local Galaxy server in two steps: 1) hard-trimming to remove 1 3’-end base (FASTQ Trimmer tool, v.1.0.0); 2) quality trimming from both ends until minimum base quality for each read ≥ 30 (FASTQ Quality Trimmer tool, v.1.0.0) [20, 21]. Reads were aligned in Galaxy using Bowtie and TopHat (TopHat for Illumina tool, v.1.5.0) [22]. Read counts per Ensembl gene ID were estimated in Galaxy using htseq-count (htseq-count tool, v.0.4.1) [23]. Sequencing, alignment, and quantitation metrics were obtained for FASTQ, BAM/SAM, and count files in Galaxy using FastQC, Picard, TopHat, Samtools, and htseq-count.

Protein coding genes were selected for further analysis. To filter for sample quality, a sample library was selected for further analysis if the fraction of unpaired reads examined compared to total FASTQ reads was >75%, median coefficient of variation of coverage was <1, and the library had >1 million reads. To filter for lowly expressed genes, genes expressed at a level of counts per million >1 in less than 3 samples were removed. Data were deposited in the GEO repository under accession number GSE69529.

### Differential expression analysis

The linear models for microarray data (Limma) R package was used to identify differentially expressed genes between HC and patients with infectious diarrhea [24, 25]. Expression counts were normalized using the TMM (Trimmed Mean of M-values) algorithm and transformed using the Limma-voom method [26]. TMM normalization generates scaling factors, taking into account both library size and the counts associated with each gene within a library. A linear model for gene expression based on group (HC or type of pathogen), patient age in years, and patient sex was used. For each gene, a t-statistic was computed using the empirical Bayes method within the Limma R package which moderates the standard deviations between genes. A false discovery rate for each gene was calculated by applying the Benjamini-Hochberg multiple testing correction to p-values calculated from t-tests. Genes with a false discovery rate of less than 0.01 and a mean expression fold-change greater than 2 relative to HC were considered to be significantly differentially expressed.

### Network analysis

Network visualization and analysis was performed using Cytoscape 3 [27]. The Reactome-FIViz App was used to form a functional interaction network of differentially expressed genes for each pathogen [28, 29]. Pathway enrichment analysis was performed on each network using the Reactome-FIViz App.

### Age and severity analysis

We calculated the median expression of differentially expressed complement genes (*C1QA*, *C1QB*, *C1QC*, and *C2*) and interferon signaling genes (*IFITM1*, *IFITM3*, *OAS2*, *OAS3*, *OASL*, *RSAD2*, *USP18*, *ISG15*, *IFIT1*, *IFIT2*, *IFIT3*, and *IRF7*). A t-test was performed to compare median gene expression differences between age or severity groups within infection categories.

### Ethics statement

Written informed consent was obtained from all children’s legal guardians to obtain clinical samples and use the data for investigational purposes. This study was approved by the Institutional Review Board at the Benaroya Research Institute, with approval number IRB10095. Samples and data were analyzed anonymously.

## Results

### Study selection

Among profiled subjects, healthy controls (HC) and patients with diarrhea disease who tested positive for rotavirus, *E*. *coli*, *Salmonella*, or *Shigella* were used for further analysis. Only patients who tested positive for the presence of a single pathogen and asymptomatic children who tested negative for the presence of all pathogens were included in this analysis. Patient samples were also screened for *Campylobacter* and *Vibrio* infection and patients that tested positive for either of these pathogens were excluded from this study. Patient samples were also selected to ensure that comparable numbers of cases and controls were sequenced from ages under and over 2. In total, 208 whole blood RNA-seq profiles were created from the dataset of controls and patients infected with a single pathogen. We further restricted the dataset to samples that had accompanying complete blood count (CBC) results (199 of the 208 samples) and filtered for RNA-seq library quality (193 of the 199 samples with CBC results). We utilized samples from 193 children (164 diarrhea cases and 29 HC, Table 1). Of these children, 101 (52%) were under two years of age, 113 (59%) were male, and 111 (67%) of the cases were within 3 days of disease onset. Clinical information including time passed since disease onset, and disease severity was also collected (Table 1, S1 Table). Of children with diarrhea, 95 (58%) were classified as mild, 48 (29%) as moderate, and 21 (13%) as severe on the basis of symptoms.

**Table 1:** Study subject demographics.

Group	Age		Sex		Days past onset		Total
	Under 2	Over 2	Male	Female	1–3 days	4–7 days	
**Healthy control**	15	14	12	17	N/A	N/A	29
***E*. *coli***	27	15	26	16	28	14	42
***Salmonella***	25	9	19	15	20	14	34
***Shigella***	11	25	22	14	26	10	36
**Rotavirus**	23	29	34	18	37	15	52
**Total**	101	92	113	80	111	53	193

### Leukocyte percentages are the primary source of transcriptional variation

**Fig 1:**
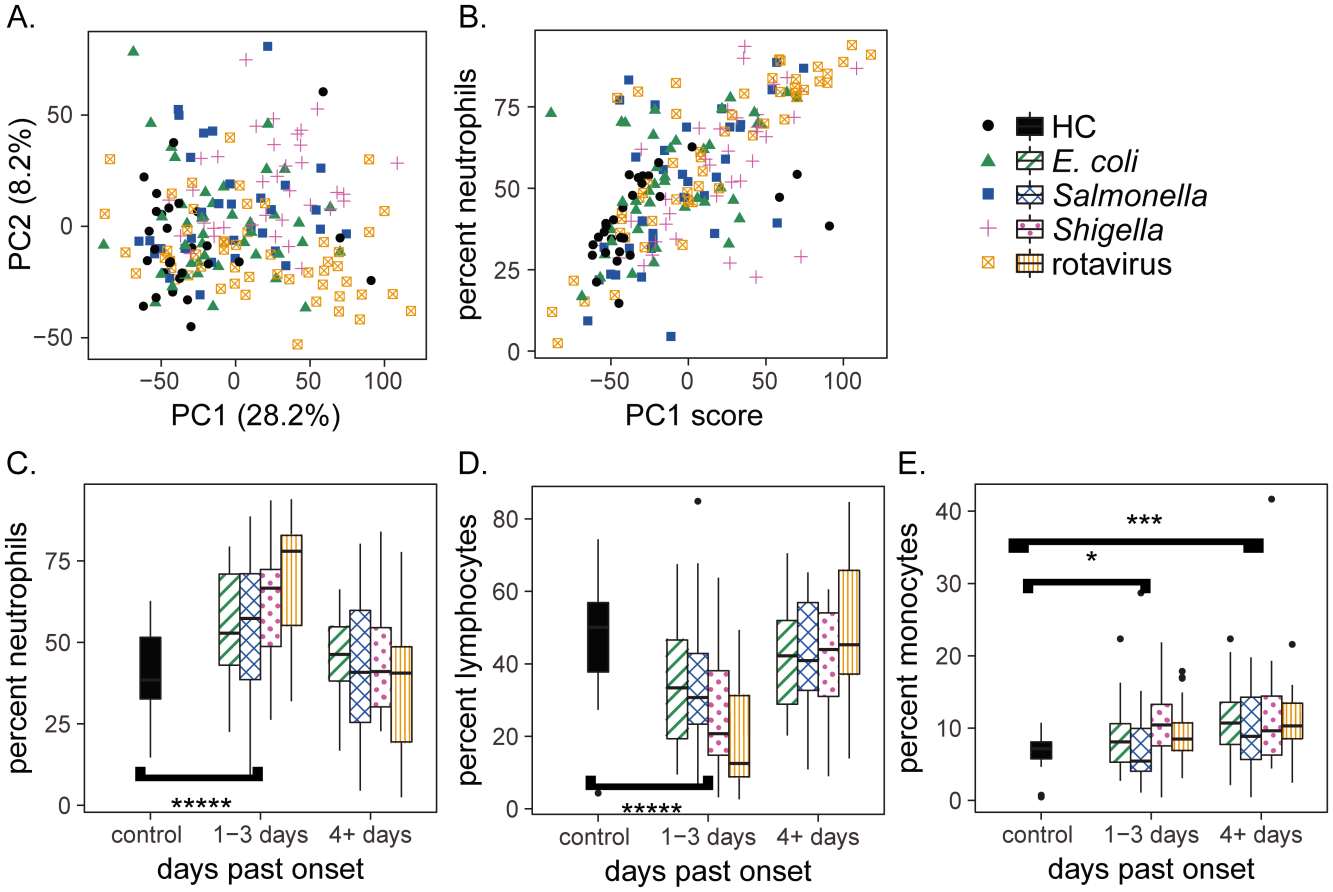
A neutrophil driven response is observed early in diarrheal disease and drives transcriptional variation. (A) Principal component analysis of transcriptional profiles from whole blood indicates clustering of HC profiles but no clustering according to infection type. (B) The first principal component is highly correlated with neutrophil percentages (r = 0.69, p < 2.2e-16). (C-E) Leukocyte subset percentages as a function of time elapsed between disease onset and blood RNA collection. * p < 0.01, *** p < 0.001, **** p < 1e-5, Wilcoxon rank-sum test (HC compared to all disease cases).

As an initial test of gene expression variation between individuals and organisms, we performed principal component analysis (PCA), a method for reducing data dimensionality while retaining most of the variation in the data set [30]. PCA analysis of our RNA-seq data demonstrated overlap of profiles from HCs and individuals infected with each of the 4 infectious agents, although most HC samples clustered with a lower first principal component (PC1) score (Fig 1A). This finding suggests global differences in host gene expression patterns in individuals with infectious diarrhea compared to HCs.

To determine whether gene expression could be linked to clinical parameters, we searched for clinical correlates of PC1, which explained 28% of the variability in the RNA-seq data. We found that neutrophil percentages and days past disease onset strongly correlated with each other (Pearson’s r = 0.69, p < 2.2e-16) and with the first principal component of variation in the RNAseq data (Pearson’s r = -0.38, p < 1e-6) (Fig 1B and S1 Fig). The strength and significance of these correlations with PC1 suggested that leukocyte subset percentages were related to the days past onset. To test for this relationship, we compared levels of neutrophils, lymphocytes and monocytes, respectively, to days past onset (Fig 1C–1E). Patients 1–3 days past disease onset had elevated neutrophil and monocyte percentages but decreased lymphocyte percentages relative to HCs, consistent with an early innate immune response to infection. Patients 4–7 days past disease onset had increased monocyte percentages relative to HCs, but similar neutrophil and lymphocyte percentages (Fig 1C–1E). Together, these findings demonstrate that cell composition differences are an important source of variation in transcript levels in whole blood host responses to the pathogens studied.

### Unique differential gene expression is observed in *Shigella* and rotavirus patients

**Fig 2:**
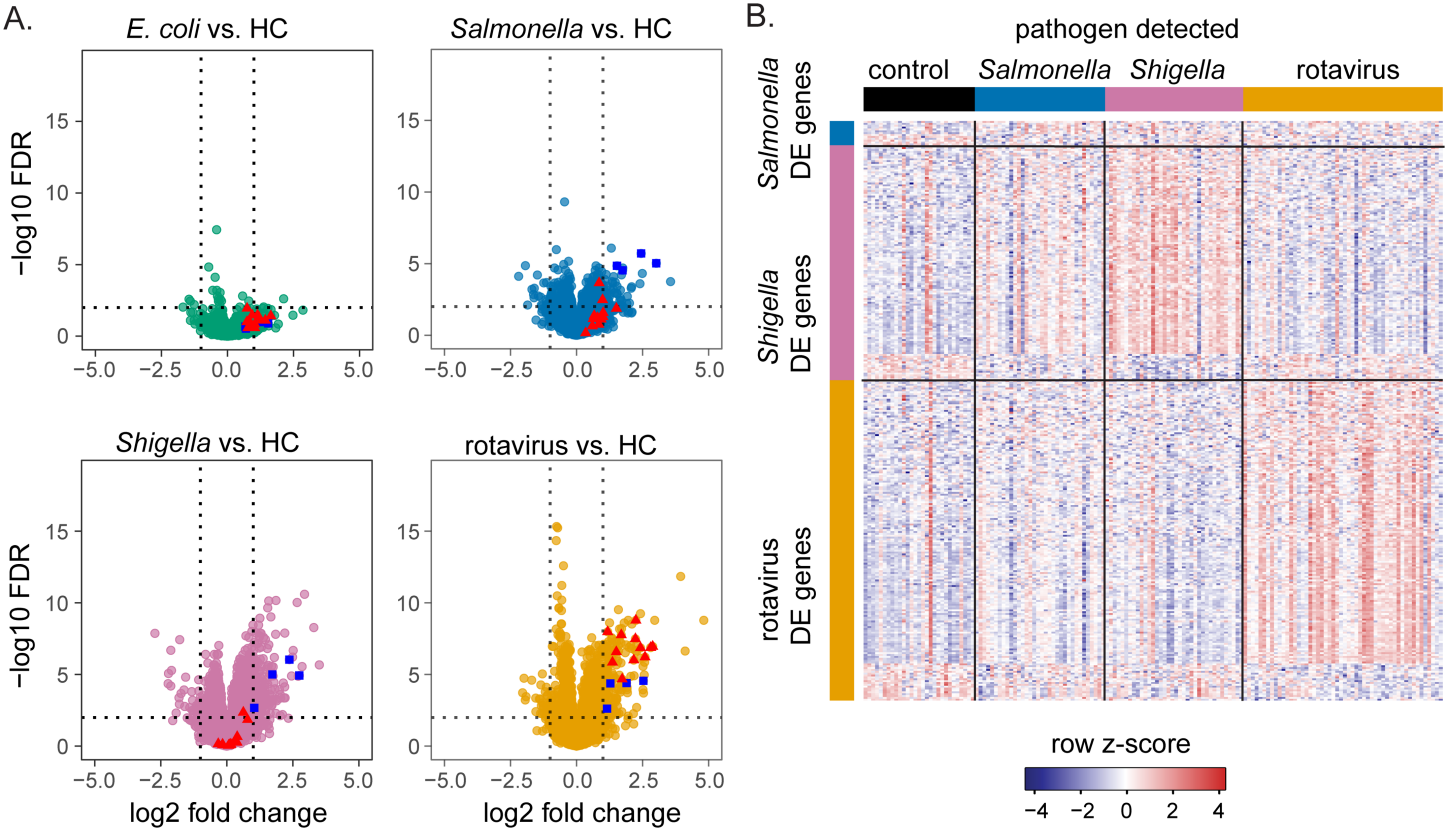
Transcriptional differences are observed between diarrheal disease groups and HC. (A) Differential gene expression between controls and each pathogen group is shown. Each point represents a gene, the dashed vertical lines indicate log2 fold-changes of -1 and +1, and the dashed horizontal line indicates a false discovery rate (FDR) of 0.01. Selected genes in the complement pathway are highlighted in blue and selected genes involved in interferon signaling are highlighted in red. B) The heatmap shows differentially expressed genes unique to each of the 3 pathogen groups. *E*. *coli* is not included as one only differentially expressed gene was unique to this group. Rows represent genes with a log fold change of less than -1 or greater than 1 and FDR of less than 0.01 that are only differentially expressed in either *Salmonella*, *Shigella*, or rotavirus patients relative to HC. Columns represent individual profiles.

To identify pathogen-specific effects on patient gene expression, we performed differential gene expression analysis. In addition to the pathogen detected, we included neutrophil and monocyte percentages in a model as an important source of gene expression differences. Since groups infected with different pathogens were not balanced according to age and sex, these variables were also included in the model. Relative to HC, the most pronounced differential gene expression differences were in the *Shigella* and rotavirus groups (Fig 2A, S2 Table). We found 184 genes were differentially expressed in the *Shigella* group, of which 126 genes were differentially expressed unique to the *Shigella* group and not differentially expressed in any other infection group relative to controls. Similarly, 213 genes (172 unique) were differentially expressed in the rotavirus group, 78 genes (15 unique) in the *Salmonella* group, and only 6 genes (1 unique) in the *E*. *coli* group. Genes with differential expression unique to the *Salmonella*, *Shigella*, or rotavirus groups are displayed in a heatmap in Fig 2B. This heatmap indicates organism-specific differences in gene expression in each of these groups relative to HC. To address the heterogeneity of *E*. *coli* pathotypes, we performed a second analysis in which infections due to *E*. *coli* were grouped according to pathotype. Of the 42 *E*. *coli* cases in our study, 18 were diffusely-adherent *E*. *coli* (DAEC), 14 were enteroaggregative *E*. *coli* (EAEC), and 10 were enteropathogenic *E*. *coli* (EPEC). We found only one gene (*IFI27*) to be differentially expressed in children with DAEC relative to healthy control, no genes were differentially expressed in EAEC infections, and 5 genes (*SLC16A14*, *HID1*, *B9D1*, *PDIA5*, *ABCB9*) were differentially expressed in EPEC infections. The small number of differentially expressed genes associated with any one *E*. *coli* pathotype indicates that there are no strong transcriptional signatures associated with these pathogens. However, with fewer than 20 subjects within any pathotype group, this analysis is relatively underpowered compared to the analysis where all subjects with *E*. *coli* infections were placed in a single group. It is possible that a subtler signature is associated with *E*. *coli* pathotypes that could be revealed in a more targeted study.

### Pathways with altered gene expression observed in *Shigella* and rotavirus cases

**Fig 3:**
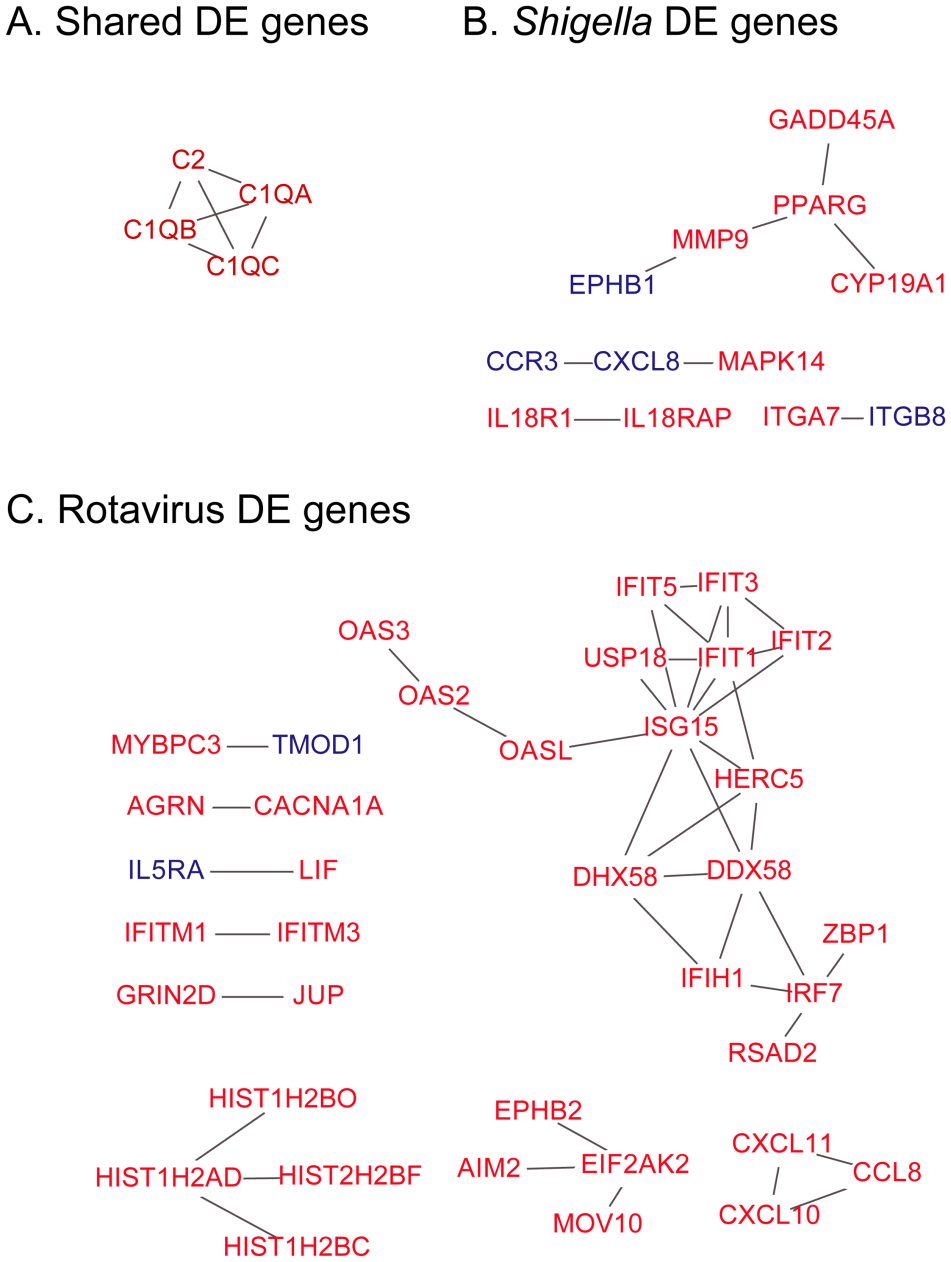
Differentially expressed genes in *Shigella* and rotavirus infections form protein-protein interaction networks. Nodes represent genes and edges represent interactions. Red nodes represent genes with increased expression relative to controls and blue nodes represent genes with decreased expression. (A-C) Shown is the network of genes differentially expressed shared by the *Salmonella*, *Shigella*, and rotavirus groups (A), genes unique to the *Shigella* group (B), and unique to the rotavirus group (C).

To gain insight into biological processes represented by host transcript responses, we performed network analysis on differentially expressed genes shared in *Salmonella*, *Shigella*, and rotavirus infections (Fig 3A) and in genes unique to each of these three groups (Fig 3B and 3C), using the Reactome database and Reactome FIViz app for Cytoscape [28, 29] (Fig 3). No interaction network was formed from genes unique to *Salmonella* infections, but interaction networks were formed from genes unique to *Shigella* and rotavirus infections (Fig 3B and 3C). Functional enrichment analysis revealed that the network of genes shared by the three groups was enriched for genes involved in the complement and coagulation cascade (FDR = 1.4e-5). The upregulated complement genes that appear in the shared network, *C1QA*, *C1QB*, *C1QC*, and *C2*, are all part of the early classical complement pathway [31] (Figs 2A and 3A). There were no biological themes enriched in genes downregulated relative to HCs.

Genes, related to neutrophil and leukocyte activation (*CD177* and *LILRA5*, respectively), cytokine and cytokine receptors (*IL10*, *IL27*, *IL18R1*, *IL5RA*, *IRAK3*), and recognition of peptidoglycan (*PGLYRP1*) and lipopolysaccharide (*LPI*) were significantly upregulated in children with *Shigella* infections (S2 Table). Genes related to inflammatory processes were also upregulated in the interaction network associated with *Shigella* infections (S2 Table, Fig 3B). These changes included increased expression of *NLRC4* and *NAIP*. These genes encode proteins which form an inflammasome that recognizes bacterial flagellin and type III secretion systems, ultimately resulting in Caspase-1 activation [32, 33]. Caspase-1 activation results in the upregulation of interleukin-1 beta and interleukin-18 [32, 33]. Expression of *IL18R1* and *IL18RAP* which encode the interleukin-18 receptor were uniquely upregulated in *Shigella* cases but not in cases due to the other pathogens, suggesting that whole blood transcript profiles of *Shigella* infected patients reflect an inflammatory host response to *Shigella* infection.

Multiple interferon response genes were observed in the network of genes upregulated in rotavirus infections (S2 Table, Fig 3C). This network was highly enriched for genes involved in interferon alpha/beta signaling (Figs 2A and 3C, FDR = 1.5e-14). Upregulated genes included multiple members of the IFN-induced protein with tetratricopeptide repeats (IFIT) family, which inhibit virus replication by binding to viral nucleic acids directly or binding to the eukaryotic translation initiation factor 3 (eIF3) and preventing the initiation of translation [34, 35]. Also included in this signature were genes involved in the OAS/RNase-L pathway and the interferon-inducible chemoattractants *CCL8*, *CXCL10*, and *CXCL11* [34–36]. *ISG15*, which encodes a ubiquitin-like protein that can be attached to multiple target proteins in a process known as ISGylation, and other genes (*HERC5*, *HERC6*, *USP18*) involved in IGSylation were upregulated as well [36].

### Complement and interferon-stimulated gene expression is unrelated to patient age

**Fig 4:**
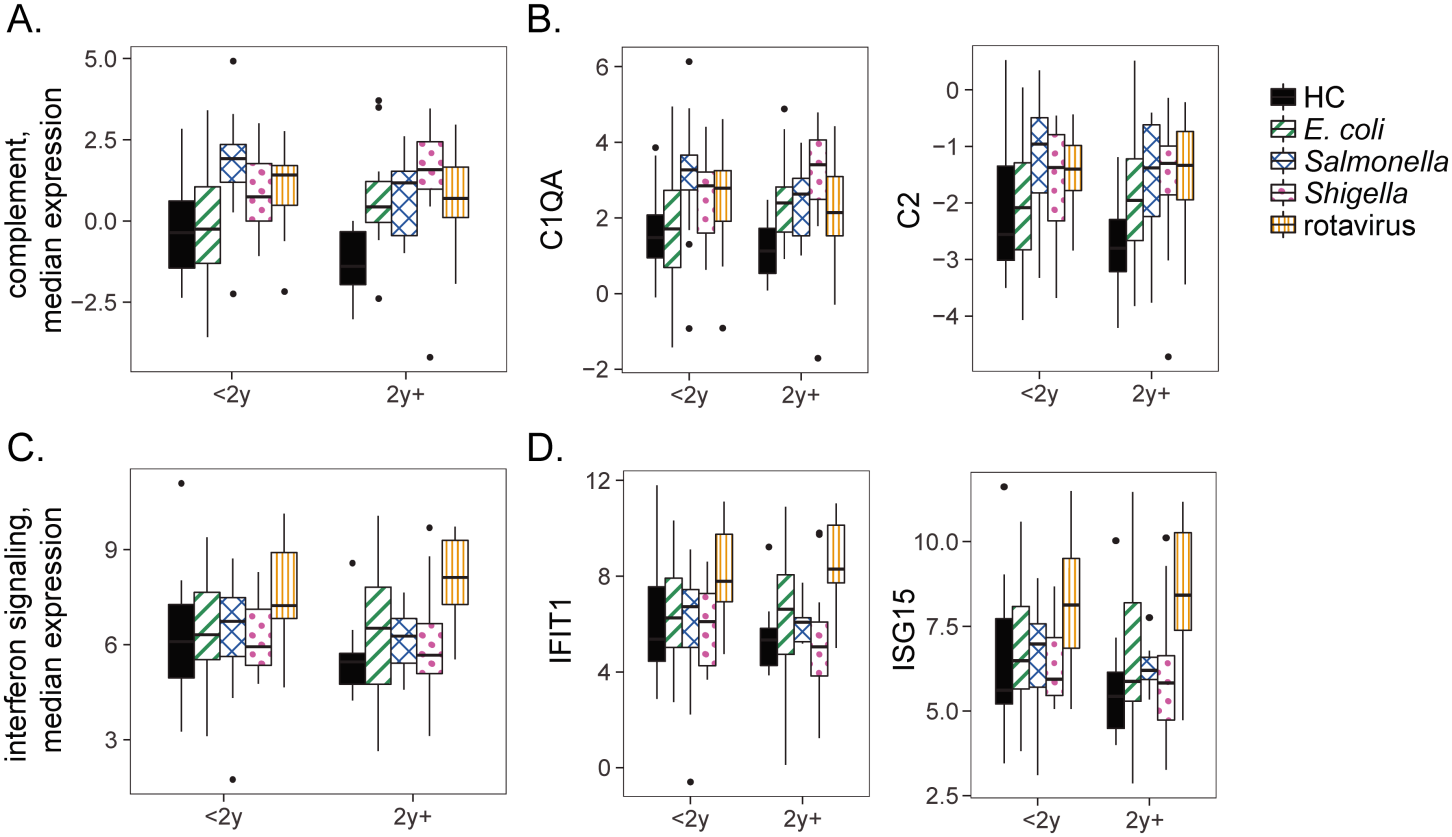
Expression of complement and interferon genes does not depend on age. (A) The median expression of differentially expressed complement genes is shown for each group stratified by age less than or greater than 2 years. (B) Expression of selected complement pathway genes. (C) The median expression of differentially expressed interferon genes is shown for each group stratified by age less than or greater than 2 years. (D) Expression of selected interferon signaling genes. X-axis: age groups; children under 2 and over 2 years of age. Y-axis: log2 residual gene expression counts after regressing out the effect of leukocyte proportions.

The patients in our dataset range from under one year old to nine years old. Significant biological changes accompany the first few years of life which could impact a patient’s response to diarrheal disease. Very young children are less likely to have had prior exposure to disease or to have completed the series of rotavirus vaccinations. Given these differences, we investigated whether complement or interferon-stimulated gene expression differed between age groups as well as between cases and controls. We stratified our cohort of children into those under 2 and over 2 years of age. After accounting for leukocyte proportions, we compared gene expression within each infection category in children under and over 2 years of age. We observed no differences in median complement or interferon-stimulated gene expression according to age (p > 0.1, t-test). Expression of representative individual interferon and complement genes by age is shown in Fig 4. These, and the other individual interferon and complement genes, show no age-associated differences in expression (p > 0.1, t-test).

### Complement and interferon gene expression are unrelated to severity, but related to cell type proportions

**Fig 5:**
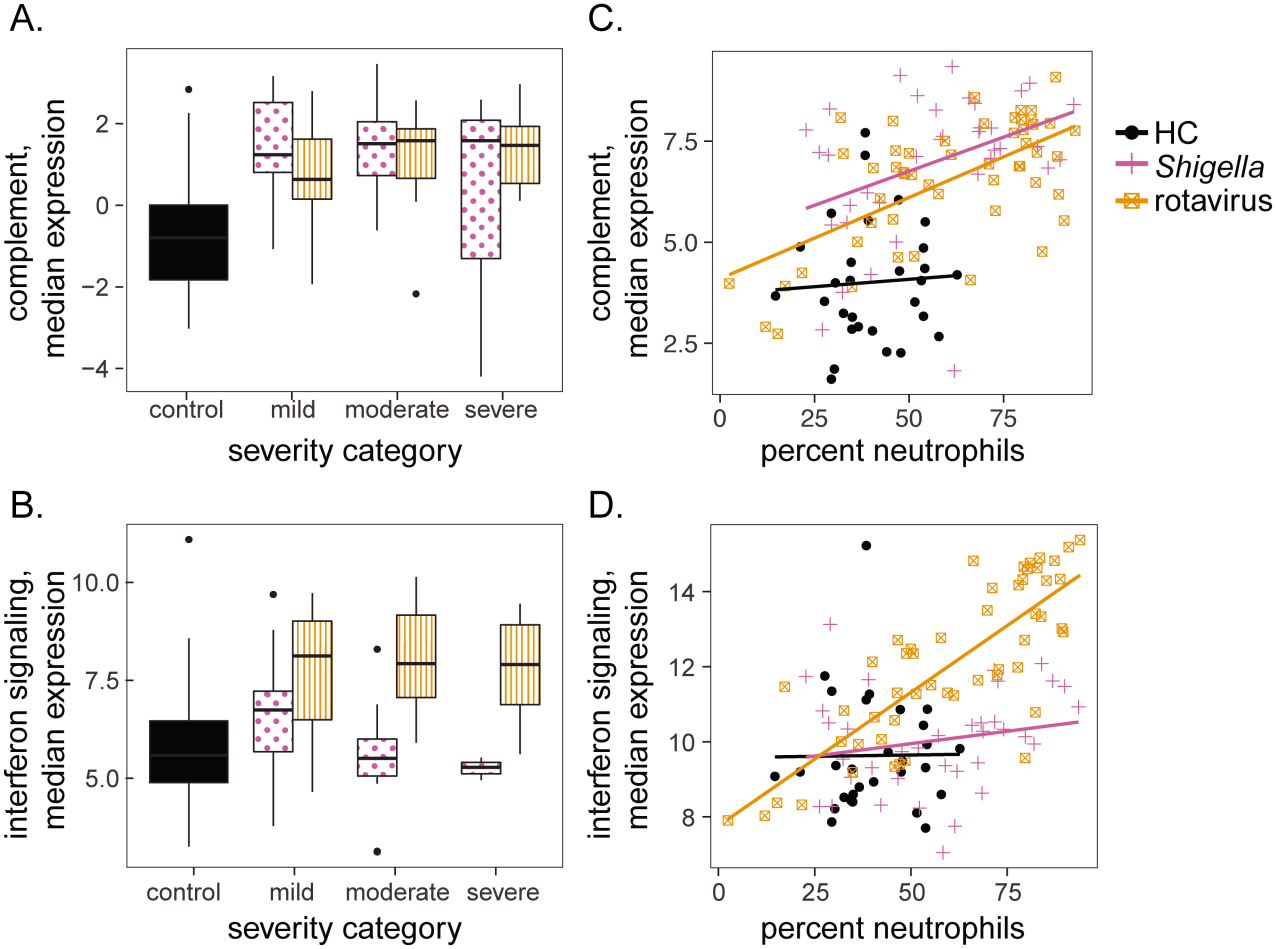
Complement and interferon gene expression were related to neutrophil counts but not to disease severity. (A and B) Complement (A) and interferon signaling (B) gene expression as a function of disease severity. Y-axis: log2 residual gene expression counts after regressing out the effect of leukocyte proportions. (C) Complement expression was positively correlated with neutrophil percentages in the rotavirus and *Shigella* groups. Y-axis: log2 gene expression counts, no correction for leukocyte differences. (D) Expression of genes involved in interferon signaling were positively correlated with neutrophil percentages in the rotavirus group. Y-axis: log2 gene expression counts, no correction for leukocyte differences.

The largest numbers of differentially expressed genes were observed in *Shigella* and rotavirus infections. To explore whether expression of the early complement pathway or interferon signaling related genes were related to disease severity in these two groups, we assigned each individual a severity category based on a modification of the World Health Organization criteria as described previously [15]. Briefly, children with no dehydration were classified as mild diarrhea, those with mild to moderate dehydration and/or bloody diarrhea were classified as moderate diarrhea, and those with hypovolemic shock and or severe electrolyte imbalance were classified as severe diarrhea. We found no relationship between disease severity and differentially expressed genes associated with complement or interferon signaling gene expression (Fig 5A and 5B). We also investigated the relationship between gene expression of these pathways and cell type proportions. In both the rotavirus and *Shigella* groups an increase in complement gene expression was accompanied by an increase in the proportion of neutrophils (Fig 5C). Complement expression was positively correlated with proportion of neutrophils in *Shigella* and rotavirus groups, but not in HC (r = 0.40, p = 0.016 for *Shigella*; r = 0.62, p = 9.1e-7 for rotavirus; r = 0.05, p = 0.77 for controls). Monocyte proportions were weakly positively correlated with complement expression in HC, but not in *Shigella* and rotavirus groups (S2 Fig, r = 0.13, p = 0.45 for *Shigella*; r = 0.08, p = 0.59 for rotavirus; r = 0.35, p = 0.07 for controls). In contrast, rotavirus, but not *Shigella* or HC groups, showed an increase in interferon response gene expression accompanied by an increase in the proportion of neutrophils (Fig 5D, r = 0.20, p = 0.24 for *Shigella*; r = 0.80, p = 8.4e-13 for rotavirus; r = 0.01, p = 0.95 for HC). This indicates that the relationship between gene expression and cell type proportions varies according to pathogen and gene function.

## Discussion

We used RNA-seq of peripheral blood to systematically evaluate the host transcript response to diarrhea-causing pathogens. In comparisons of signatures between these groups, we found that leukocyte subset proportions were the primary source of host transcript variation. Thus, development of diagnostics based on whole blood gene signatures associated with these diseases should include consideration of cell type proportions. We also found that cell type proportions were related to time since symptom onset. Neutrophil counts were higher earlier in disease, whereas lymphocytes became elevated later. This is consistent with an early, innate immune response in patients, followed by an adaptive response after several days of infection.

Our findings increase our understanding of host-pathogen interactions in diarrheal illnesses. Although it is well established that rotavirus induces an interferon response in intestinal epithelial cells, less is known about systemic interferon response to rotavirus [37, 38]. Increased expression of alpha and beta interferons has been noted in peripheral blood mononuclear cells of children with rotavirus infection [39]. A broad-based interferon response signature has been observed from blood samples of patients with other disease such as influenza and tuberculosis, but to our knowledge, an interferon response signature in whole blood of rotavirus patients has not been previously reported [12, 14]. Complement is well known to play an important role in the immune response to many pathogens. However, upregulation of early classical complement genes has not previously been observed in *Salmonella*, *Shigella*, and rotavirus infections [31, 40].

It is also noteworthy that *Shigella* and rotavirus infections had both the highest total monocyte proportions and highest early classical complement pathway gene expression, but that expression of complement genes in these patients was not correlated with proportions of monocytes. Although most complement is produced by the liver, monocytes also produce complement [41, 42]. The lack of a correlation we observed suggests a decoupling of early complement mRNA levels from monocyte proportions in blood. In contrast, we did observe a strong correlation between neutrophil levels and complement mRNA expression. Neutrophils are a well-known site of complement activity, but they are not generally thought to be a major source of complement proteins [41]. Further experimentation would be needed to assign production of complement to any cell type, the correlations we have observed lead us to hypothesize that that neutrophils may regulate levels of complement expression in diarrheal illnesses.

By profiling multiple pathogens, we learned about the utility of gene expression in peripheral blood for distinguishing pathogens with similar symptomology. For other pathogens other than *E*. *coli*, we were able to identify specific genes associated with each infection. Our finding suggests these genes could be evaluated for accuracy as biomarkers for identification of the causative pathogen in infectious diarrhea. We also discovered limitations on the value of these signatures. For example, we observed no relationship between gene expression signatures and disease severity after accounting for differences in leukocyte proportions. We also detected no age-related differences in early complement and interferon response gene expression. This was surprising given that age has previously been associated with clinical symptoms [3]. Tracking serial infections over time while stratifying for age and severity in a future study could offer finer resolution of the interactions between age, severity, infection type and host response. The scope of our study was limited to examining the host response to infection by four pathogens, but other pathogens such as *Campylobacter jejuni*, the parasites *Giardia lamblia* and *Cryptosporidium* also contribute to childhood diarrheal disease [1]. It is possible that the transcriptional pathways we report here are shared with these or other species that were outside of the scope of this study.

Although we studied patients with disease ranging from mild to severe, the patients in our study had been sought care for diarrheal disease and likely represent a more severe subset of cases than would be found in the general population. However, the gene signatures we observed did not depend on disease severity, which suggests that they would be broadly applicable. Even if confined to the context of hospitalized patients these signatures offer value. In cases requiring hospitalization, they could be used to develop tests that inform the decision-making process to determine care for the sickest patients. Currently, cost considerations are a barrier to the adoption of RNA-seq assays as a rapid diagnostic in areas with limited resources. However, future innovations in generating inexpensive microfluidics “lab on a chip” approaches, combined with decreasing sequencing costs could make transcriptional profiling accessible to clinicians and hospitals with limited resources in the future [43–46].

The implication of host interferon responses in rotavirus, inflammasome activation in *Shigella*, and complement in *Salmonella*, *Shigella*, and rotavirus indicates the ability of blood profiling to reflect the pathogenesis of these diseases. We chose to focus on a subset of possible diarrhea causing pathogens. However, blood profiling is a broadly applicable approach that can be used to simultaneously study multiple pathogens associated with various diseases and offers the potential to run multiple diagnostic tests from collection of a single sample.

## Supporting information

S1 Fig Relationship between PC1 and days past disease onset. (A) Principal component analysis, colored by days past disease onset. (B) PC1 values according to days past disease onset.
(TIF)

S2 Fig Monocyte proportions and complement expression. Median complement gene expression as a function of the proportion of monocytes.
(TIF)

S1 Table Clinical information and complete blood count data. (XLSX)

S2 Table Differentially expressed gene lists. (XLSX)
