# Supplementary figures and images for: Shared and organism-specific host responses to childhood diarrheal diseases revealed by whole blood transcript profiling
Source: PLoS One. 2018 Jan 29;13(1):e0192082. doi: 10.1371/journal.pone.0192082 (PMC5788382; doi:10.1371/journal.pone.0192082)

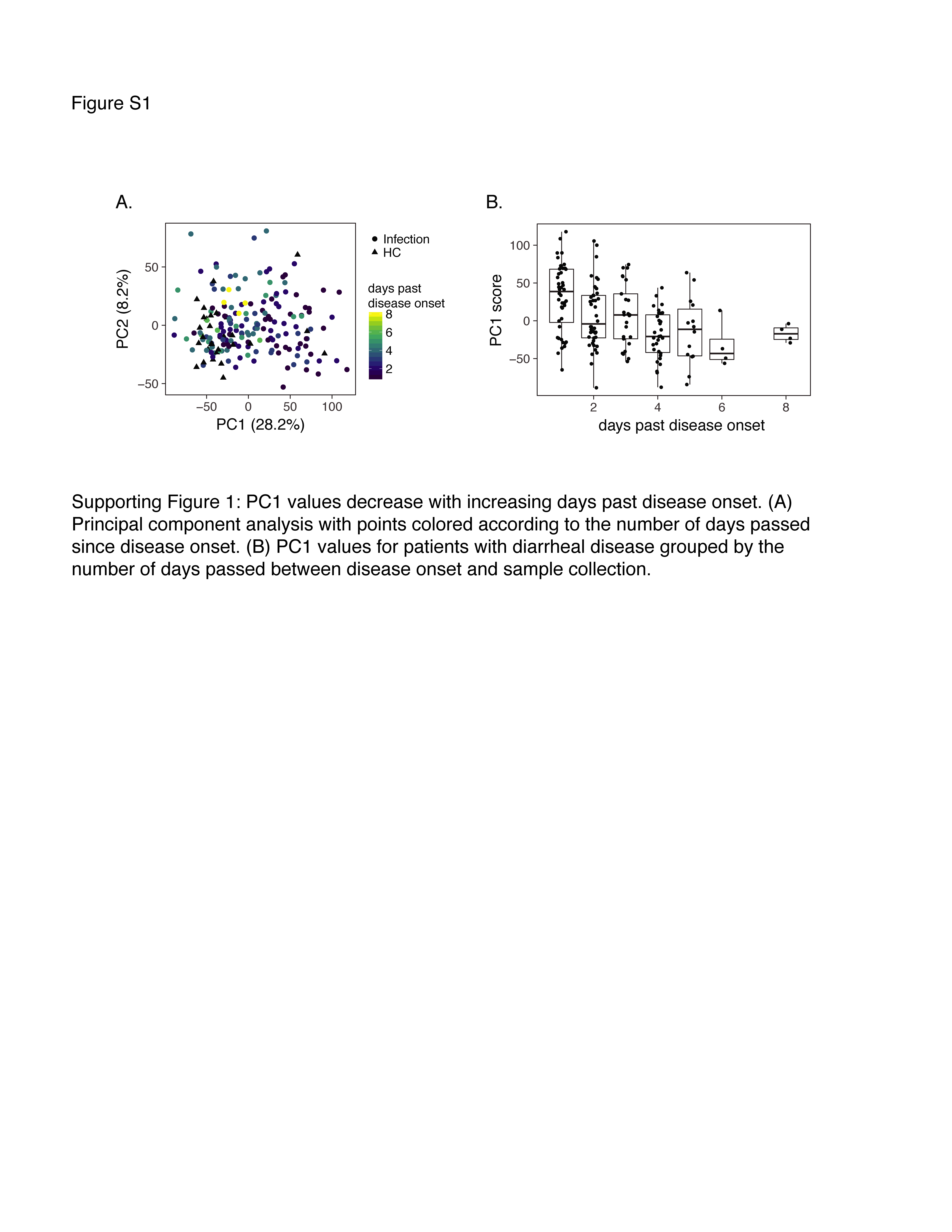

Supplement: S1 Fig — (A) Principal component analysis, colored by days past disease onset. (B) PC1 values according to days past disease onset. (TIF) [file pone.0192082.s001.tif]

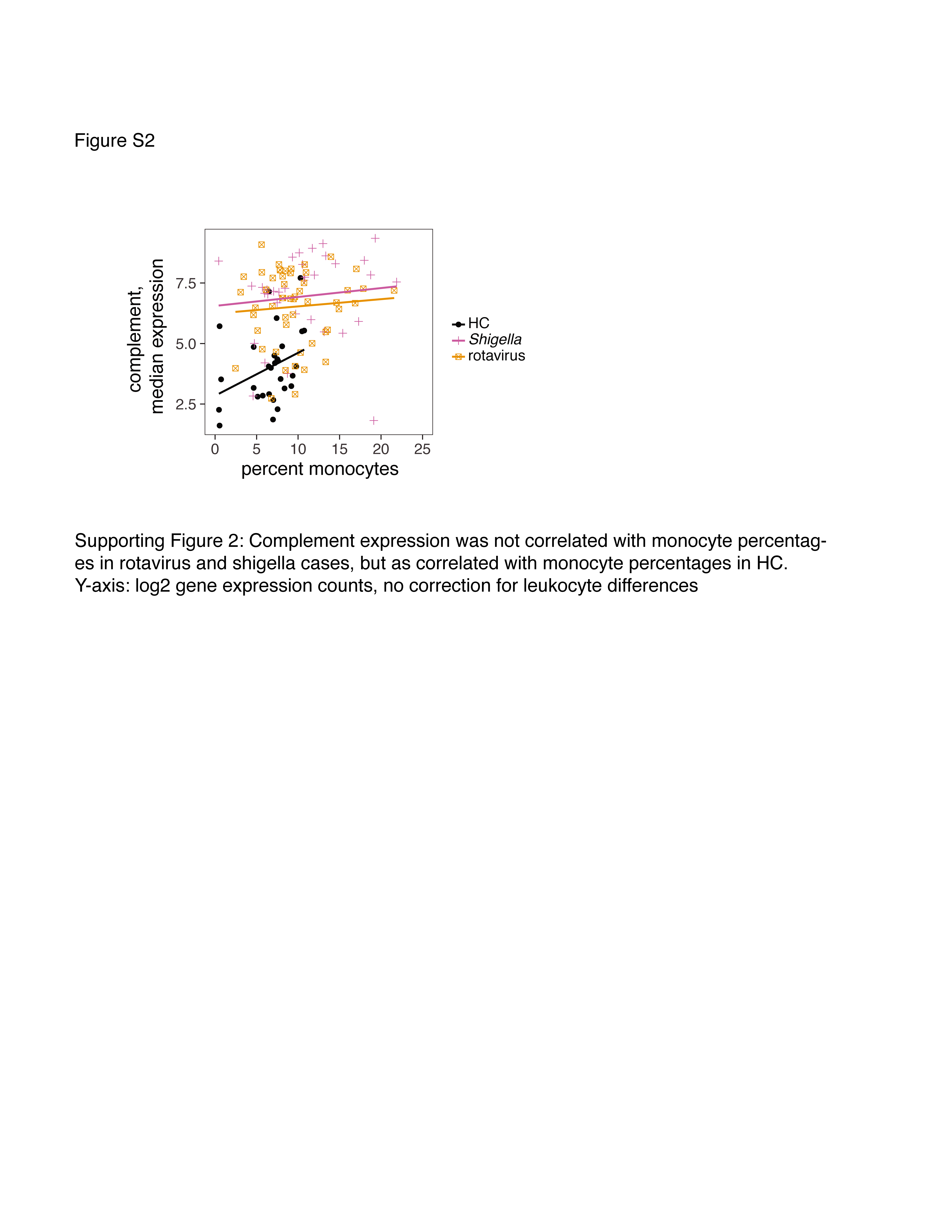

Supplement: S2 Fig — Median complement gene expression as a function of the proportion of monocytes. (TIF) [file pone.0192082.s002.tif]
